# Improving breast cancer treatments using pharmacomicrobiomics

**DOI:** 10.1128/mbio.03422-24

**Published:** 2025-01-17

**Authors:** Aswin Anand Pai, Aadra Prashant Bhatt

**Affiliations:** 1 Department of Haematology, Christian Medical College, Vellore, India; 2 Center for Gastrointestinal Biology and Disease and Department of Medicine, Division of Gastroenterology and Hepatology, University of North Carolina at Chapel Hill, Chapel Hill, North Carolina, USA; Northern Arizona University, Flagstaff, Arizona, USA

**Keywords:** microbiome, precision medicine, pharmacomicrobiomics

## Abstract

Tamoxifen is the mainstay treatment for estrogen-positive breast cancer for over half a century. However, a significant proportion of patients experience disease recurrence due to treatment failure attributed to various factors, including disease pathology, genetics, and drug metabolism. Alam et al. introduce gut microbiota as a key factor influencing tamoxifen pharmacokinetics (Y. Alam, S. Hakopian, L. Ortiz de Ora, I. Tamburini, et al., mBio 16:e01679-24, 2024, https://doi.org/10.1128/mbio.01679-24). The authors present compelling evidence that functional differences in the gut microbiota, specifically the bacterial enzyme β-glucuronidase, leads to inter-individual variability in systemic exposure of tamoxifen, affecting drug efficacy. This study provides novel insights into the impact of the gut microbiota on tamoxifen pharmacokinetics, the latest example of how pharmacomicrobiomics, or the study of drug-microbe interactions, can enhance precision medicine for numerous diseases.

## COMMENTARY

Since the late 1960s, tamoxifen (TAM) has revolutionized the treatment landscape for estrogen-positive breast cancer (BC). The 10-year disease recurrence rate for patients using TAM is around 30% (1, 2), suggesting a high degree of inter-individual variability in treatment response. Differential gene expression of drug metabolizing enzymes is partially responsible for TAM’s variable therapeutic efficacy: TAM metabolism involves multiple step-wise, enzyme-catalyzed biotransformations that convert the prodrug TAM to its active metabolites (*Z*)-4-hydroxytamoxifen (4HT) and (*Z*)-endoxifen (END), each with significantly greater antiestrogenic potency than TAM (3–5). 4HT and END levels vary in the plasma, resulting from pharmacogenetic variations in hepatic drug metabolism enzymes, notably cytochrome P450 and UDP-glucuronosyltransferases that convert 4HT and END into their inactive glucuronide conjugates. These are subsequently excreted via the bile into the gut, where they are hydrolyzed, and the majority reabsorbed via enterohepatic circulation to reenter systemic circulation to continue exerting anticancer effects. Intestinal disposition of TAM metabolite glucuronides leads to encounters with the gut microbiota, many of which encode an enzyme called β-glucuronidase (GUS), known to hydrolyze and reactivate multiple xenobiotic and endobiotic substances (6, 7).

There are limited, isolated reports of the importance of the microbiome on BC and TAM exposure. A recent study by Li et al. (8) employing an MCF-7 xenograft mouse model of BC found that TAM treatment altered gut microbiota composition, enriching taxa positively correlated with inflammation, including *Prevotellaceae_UCG_001* and *Akkermansia*; concomitant upregulation of inflammatory cytokines such as IL-6, IFN-γ, and IL12P70 suggested a state of inflammation post-TAM exposure. Another recent study by Diot et al. (9) using a *C. elegans* model discovered that different bacteria contribute to variable TAM toxicity via distinct bacterial mechanisms. These reports not only implicate the role of intestinal bacteria in mediating TAM toxicity but also provide some mechanistic clues.

In a recent study published in *mBio*, Alam et al. (10) aimed to study the role of gut microbiome on TAM PK. Alam and colleagues hypothesized that bacterial β-glucuronidases perform this hydrolysis reaction, thereby contributing to the variable pharmacokinetics (PK) of TAM. Using gnotobiotic- and antibiotics-treated mouse models, the authors demonstrated that gut bacteria and prolonged exposure to TAM significantly influence TAM PK. Blood TAM levels were significantly higher in humanized mice treated daily with TAM, compared to germ-free mice treated with TAM or humanized mice treated with vehicle alone. These findings were corroborated by depleting the microbiota of specific pathogen-free mice with antibiotics followed by TAM administration; mice had lower peak TAM levels and reduced levels of the active TAM metabolite 4HT. Collectively, these findings implicate gut bacteria alteration of TAM PK.

16S rRNA gene amplicon analysis of gnotobiotic mice revealed minimal compositional changes following TAM treatment. No differences in microbial alpha diversity or abundance could account for variability in TAM PK, suggesting an underlying variability in specific bacterial function and not composition. Metabolomic analyses of cecal content of humanized gnotobiotic mice uncovered differences in several putative metabolites following TAM exposure. Based on these murine models, the authors established that the gut microbiota influences TAM PK.

The authors performed a correlation analysis between bacterial taxa and ^13^C-TAM exposure, to attempt attributing specific bacterial taxa with systemic TAM levels. Although no significant correlation was found between bacterial taxa and ^13^C-TAM AUC, the authors observed that Clostridia (*Firmicutes*) and Erysipelotrichia (*Firmicutes*) significantly correlated with peak ^13^C-TAM levels. Interestingly, many members of Clostridia encode GUS activity (11–13).

To determine the extent of inter-individual variability in TAM metabolism in humans, the authors cultured fecal bacteria from healthy human donors with a physiologically relevant dose (60 µM) of TAM or vehicle. *In fimo* (14) metabolomics (both untargeted and targeted) revealed fatty acid dysregulation following TAM exposure in aerobic or anaerobic conditions, suggesting alterations in bacterial lipid metabolism possibly due to the hydrophobicity of TAM. Furthermore, using untargeted metabolomics, the authors observed several unknown TAM-derived metabolites (e.g., hydroxylated or potentially glycosylated TAM), which could serve as potential fecal biomarkers of TAM response. Altogether, the metabolomics data highlights the importance of functional assessment of the gut microbiome.

Glucuronidation is the primary metabolic pathway for the host metabolism of TAM. The end recycling of glucuronidated TAM metabolites [4HT-G and (*Z*)-endoxifen (END-G)] from the intestine to blood, mediated by bacterial β-GUS, is crucial for achieving optimal therapeutic blood levels of TAM. To investigate the role of bacterial β-GUS, the authors quantified *in fimo* hydrolysis of 4HT-G and END-G using crude lysates prepared from feces of nine donors. Notably, 4HT-G was consistently and efficiently hydrolyzed (90 minutes post-incubation) by β-GUS across all donors, implying a uniform activity. In contrast, END-G hydrolysis exhibited significant variability, with one donor displaying the highest hydrolysis. These findings further confirm known variation in substrate specificities for bacterial β-glucuronidases (6, 15).

To delineate the inter-individual variability in END-G hydrolysis, the authors then examined every donor’s fecal sample and their respective microbial GUS genes. Using whole-genome shotgun sequencing, the authors observed *in fimo* END-G hydrolysis significantly and positively correlated with the abundance of two *Bacteroides fragilis* GUS genes that are essentially identical, differing only by two amino acids. These findings highlight the importance of interindividual variations of gut microbiota function, here β-GUS activity, contributing to drug responses. Such functional variations thus play a pivotal role in achieving optimal therapeutic TAM exposure, which could, in turn, improve outcomes in patients with BC. Thus, systematically examining drug-microbiota interactions, or pharmacomicrobiomics, could improve drug responses in a variety of contexts.

In summary, this pioneering study by Alam et al. provides a novel mechanism through which the function of gut microbiota influence TAM PK. The authors elegantly elucidated the interplay between the gut microbiome in terms of bacterial β-GUS activity and the metabolism of TAM. Furthermore, they ascribe inter-individual variability in β-GUS activity contributing to systemic exposure of TAM, which is crucial for exerting anticancer effects. In the future, it will be interesting to examine the relative contribution of bacterial β-glucuronidase in patients with differential treatment responses to TAM. In conclusion, unraveling the role of the gut microbiota in TAM metabolism opens the door to the novel field of pharmacomicrobiomics to individualize TAM-based hormone therapy for BC.
